# Combined Aerobic–Resistance Training and Taurine Supplementation Reduce Asprosin and Elevate Spexin in Men with Obesity: A 12-Week Supplement-Blinded, Randomized Controlled Trial

**DOI:** 10.3390/nu18142325

**Published:** 2026-07-16

**Authors:** Saber Saedmocheshi, Wissem Dhahbi, Ayoub Saeidi, Amir Rahmani

**Affiliations:** 1 Department of Physical Education and Sport Sciences, Faculty of Humanities and Social Sciences, University of Kurdistan, Sanandaj 66177-15175, Iran; 2 Research Unit “Sport Sciences, Health and Movement”, High Institute of Sports and Physical Education of Kef, University of Jendouba, Kef 7100, Tunisia; wissem.dhahbi@gmail.com; 3 Training Department, Police College, Qatar Police Academy, Doha 7157, Qatar

**Keywords:** adiponectin, energy metabolism, gluconeogenesis, hypothalamus, insulin resistance, metabolic syndrome, myokines, oxidative stress

## Abstract

Aim: Asprosin, a white adipose tissue-derived glucogenic adipokine, and spexin, a satiety-promoting neuropeptide, are dysregulated in obesity, yet their collective modulation by structured exercise and nutritional supplementation remains poorly characterized. This trial investigated the effects of 12 weeks of combined aerobic–resistance training, with and without taurine supplementation, on plasma asprosin, spexin, and body composition in obese men. Methods: Forty-four obese men (BMI ≥ 30 kg/m^2^) were allocated in a randomized, placebo-controlled, supplement-blinded design to control plus placebo (CON), taurine supplementation alone (SUP; 3 g/day), exercise plus placebo (EX), or exercise plus taurine (EX + SUP; n = 11 per group). The 12-week protocol combined aerobic training at 60% heart rate reserve (Karvonen formula) with progressive resistance training at 60% of one-repetition maximum, with load re-estimated every four weeks. Plasma asprosin and spexin were quantified by ELISA; body fat percentage (BFP) was assessed by bioelectrical impedance analysis. Two-way repeated-measures ANOVA with Bonferroni post hoc correction and Cohen’s d effect size estimation were applied throughout. Results: Significant Group × Time interactions were detected for all five outcomes (all *p* < 0.05). EX + SUP generated the greatest reductions in plasma asprosin (Δ = −11.20 ng/mL, *p* < 0.001), body mass (Δ = −5.50 kg, *p* = 0.003), BMI (Δ = −1.80 kg/m^2^, *p* = 0.002), and BFP (Δ = −4.90%, *p* < 0.001), together with the greatest elevation in plasma spexin (Δ = +0.29 ng/mL, *p* < 0.001); effect sizes were large across all EX + SUP outcomes (Cohen’s d = 1.17 to ≥1.87). CON showed no significant change in any variable (all *p* > 0.05). Conclusions: Combined aerobic–resistance training with taurine supplementation produced statistically superior, large-magnitude improvements in adipokine regulation and body composition relative to either modality in isolation, demonstrating greater improvements in the combined intervention group compared with either modality applied independently. Given the per-group sample of n = 11, these findings should be considered preliminary and exploratory; independent replication in larger, adequately powered trials is required before any clinical translation can be considered.

## 1. Introduction

Obesity is now recognized as a chronic metabolic disease characterized by systemic low-grade inflammation, progressive adipokine dysregulation, and deteriorating insulin signaling. Aging and metabolic dysfunction strongly exacerbate the pathophysiological progression of obesity [1], while data from the National Health and Nutrition Examination Survey confirm that obesity currently affects 40.3% of US adults, with severe obesity reaching 9.4% of this population [2]. Excess adipose tissue in the obese state functions as a dysregulated endocrine organ, secreting pro-inflammatory cytokines, chemokines, and adipokines that collectively disrupt glucose homeostasis, promote insulin resistance, and accelerate the clinical progression of type 2 diabetes mellitus, dyslipidemia, and cardiovascular disease. This pathophysiological continuum is substantially mediated by the dysregulated secretion of recently characterized adipokines, among which asprosin and spexin have attracted considerable scientific attention as mechanistically distinct but functionally complementary regulators of energy balance [3,4,5].

Asprosin is a 30 kDa glucogenic adipokine identified in 2016 as the C-terminal cleavage product of profibrillin-1, encoded by exons 65 and 66 of the FBN1 gene and released principally by white adipose tissue under fasting conditions [4]. Following its entry into systemic circulation, asprosin acts on hepatocytes via the olfactory receptor OLFR734, activating the G protein-cAMP-PKA signaling cascade to stimulate hepatic glucose production, a mechanism functionally analogous to glucagon [4]. Asprosin concurrently crosses the blood–brain barrier and activates hypothalamic AgRP neurons, thereby stimulating appetite and establishing a dual orexigenic-glucogenic axis that amplifies positive energy balance. Critically, circulating asprosin concentrations are pathologically elevated in obesity, with plasma levels reported at up to four times those of normal-weight individuals, and excess asprosin impairs skeletal muscle insulin signalling via the PKCδ/endoplasmic reticulum stress pathway [6]. These properties position asprosin as both a mechanistic driver of obesity-associated metabolic deterioration and a clinically relevant target for intervention.

Spexin, also designated neuropeptide Q, is a 14-amino acid bioactive peptide encoded by the Ch12:orf39 gene and expressed across a broad spectrum of central and peripheral tissues, including the hypothalamus, white adipose tissue, liver, kidney, and pancreas. Spexin operates as a counter-regulatory signal to positive energy balance: it suppresses food intake through GalR2/GalR3 receptor-mediated inhibition of NPY/AgRP hypothalamic neurons, inhibits long-chain fatty acid uptake into adipocytes, and modulates galanin-receptor-dependent lipolysis, with recent evidence implicating it in white adipose tissue thermogenic regulation through the JAK2-STAT3 pathway [5,7]. In the obese state, SPX expression is transcriptionally suppressed by adipocyte lipid overload and chronic inflammatory signaling, producing plasma spexin concentrations significantly lower than those observed in normal-weight individuals (0.43 ± 0.11 vs. 0.61 ± 0.23 ng/mL, *p* < 0.001; [5]). This suppression reinforces the orexigenic milieu by relieving a primary satiety signal, making spexin restoration a coherent therapeutic objective in obesity management.

Combined aerobic–resistance exercise training represents the most extensively validated non-pharmacological strategy for correcting obesity-related adipokine dysregulation. A 2025 systematic review and meta-analysis by Rahimi et al. [6], encompassing 14 randomized controlled trials, confirmed that exercise training significantly reduces circulating asprosin with a pooled Hedges’ g of −1.70 (95% CI [−2.17, −1.23], *p* = 0.0001), with the magnitude of effect modulated by training modality, intervention duration, and baseline adiposity. Within this context, concurrent aerobic–resistance protocols targeting both oxidative and mechanical metabolic pathways appear to produce the most robust adipokine adaptation: Suder et al. [8], demonstrated in a 12-week randomized controlled trial involving males with metabolic syndrome that combined training produced significantly greater asprosin reductions than aerobic training performed in isolation, a result attributed to superior visceral fat mobilization and amplified myokine-mediated suppression of adipokine secretion. Similarly, exercise-induced spexin elevations have been documented exclusively in responders demonstrating concurrent improvements in cardiorespiratory fitness, implicating aerobic capacity as a requisite condition for full neuroendocrine benefit [5]. Despite this evidence base, the extent to which nutritional adjuvants may potentiate exercise-mediated adipokine co-regulation in obese populations remains poorly defined.

Taurine (2-aminoethanesulfonic acid) is an endogenous sulfur-containing amino acid with established antioxidant, anti-inflammatory, and lipid-regulatory properties, circulating concentrations of which are reduced in the obese state. A meta-analysis encompassing nine randomized controlled trials confirmed that long-term taurine supplementation significantly reduces triglycerides (WMD = −0.56 mg/dL, *p* = 0.002), total cholesterol (WMD = −0.71 mg/dL, *p* = 0.002), and fasting insulin (WMD = −2.15 µU/mL, *p* = 0.0001) in overweight and obese adults [9]. Its capacity to upregulate PGC-1α and PPARγ expression, shift adipose tissue macrophage polarization from the pro-inflammatory M1 to the anti-inflammatory M2 phenotype, and attenuate reactive oxygen species production in hypertrophied adipocytes provides a plausible molecular substrate for augmenting exercise-induced adipokine responses. Preliminary evidence from combined exercise and taurine protocols in obese women confirms greater cytokine modulation and improved subcutaneous white adipose tissue plasticity than exercise alone [10], yet no controlled trial has specifically examined the co-effects of combined training and taurine co-supplementation on the parallel regulation of asprosin and spexin in obese men.

Accordingly, the present trial aimed to determine whether 12 weeks of combined aerobic–resistance training supplemented with taurine (3 g/day) produces superior modulation of plasma asprosin, plasma spexin, and body composition indices compared with exercise alone, supplementation alone, or a control condition in obese adult males.

## 2. Methods

### 2.1. Participants

This randomized, placebo-controlled, supplement-blinded trial enrolled 44 sedentary obese men (BMI ≥ 30 kg/m^2^; waist-to-height ratio > 0.5) aged 23 to 32 years, recruited via public announcements in administrative and community centers. Inclusion required the absence of regular physical activity for a minimum of six months prior to enrollment, no history of cardiovascular, hepatic, renal, or metabolic disease (including type 2 diabetes), no current pharmacological treatment, and no musculoskeletal injury contraindicated for exercise participation. All candidates underwent pre-participation cardiovascular screening performed by a certified cardiologist. Individuals reporting alcohol or substance use were excluded.

**Figure 1:**
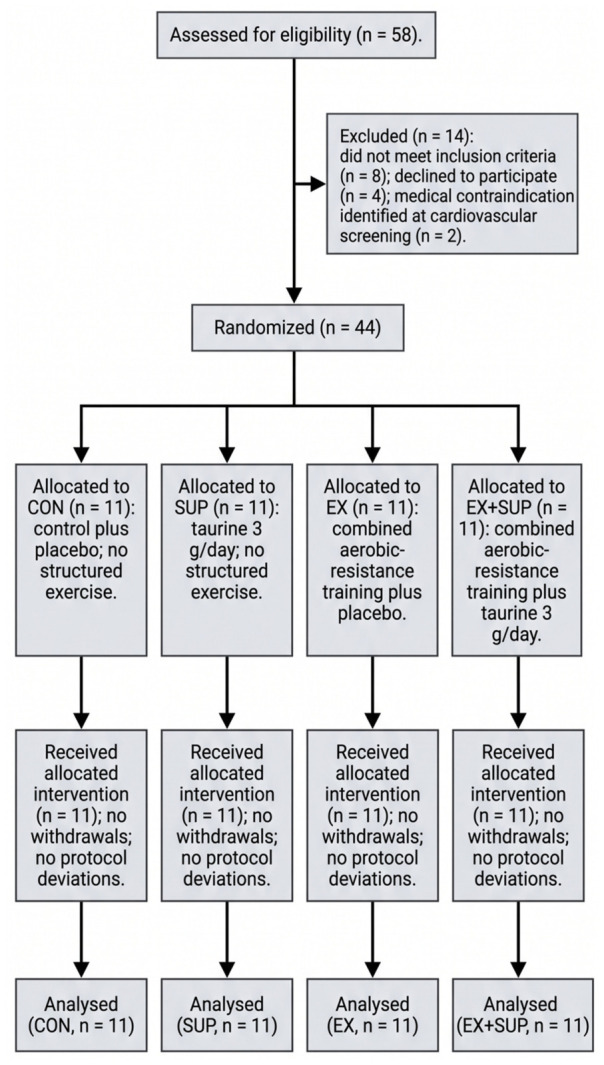
CONSORT 2010 participant flow diagram. The figure documents the complete screening, allocation, follow-up, and analysis flow for all four experimental groups. Zero attrition was recorded across the 12-week intervention period. CON = control plus placebo; EX = combined aerobic–resistance exercise plus placebo; EX + SUP = combined aerobic–resistance exercise plus taurine supplementation; SUP = taurine supplementation plus sham exercise.

A priori sample size estimation was conducted using G*Power 3.1 [11], based on a two-way repeated-measures ANOVA design, an anticipated effect size of f = 0.40 (large; derived from comparable combined exercise and supplementation trials in obese populations: [12,13]), a statistical power of 0.80, and a significance level of α = 0.05. This calculation indicated a minimum of 10 participants per group; 11 participants per group (total n = 44) were enrolled to account for potential attrition. Participants were allocated to one of four groups: (1) exercise + placebo (EX, n = 11), (2) taurine supplementation + no exercise (SUP, n = 11), (3) exercise + taurine supplementation (EX + SUP, n = 11), and (4) control + placebo (CON, n = 11). Allocation was performed by a statistician not involved in data collection or training delivery, using a computer-generated randomization sequence, with concealment maintained via sequentially numbered, opaque sealed envelopes. All participants provided written informed consent after receiving a full explanation of study procedures, potential risks, and their right to withdraw at any time without consequence. The study was conducted in accordance with the Declaration of Helsinki and received ethics approval from the Institutional Committee of the University of Kurdistan (IR.HSU.REC.1404.042; approval date: 25 October 2025). The trial was prospectively registered in the Iranian Registry of Clinical Trials (IRCT20120129008863N13; approval date: 17 November 2025) prior to participant enrollment (Figure 1).

### 2.2. Study Design

This study employed a pre-test/post-test, parallel-group, randomized controlled design over 12 weeks [14]. The four-group structure was selected to permit independent and interactive assessment of exercise training and taurine supplementation effects, consistent with factorial designs employed in comparable obesity intervention research [15,16]. Baseline anthropometric and biochemical assessments were completed during the week preceding the intervention. Post-intervention assessments were conducted 48 h after the final training session to allow acute exercise effects to dissipate while preserving chronic training adaptations.

Supplement blinding was maintained by encapsulating taurine and placebo (corn starch) into identical gelatin capsules matched for size, color, and appearance, prepared and dispensed by an independent pharmacist not affiliated with the research team. Participants and laboratory analysts performing biochemical assays were unaware of capsule content throughout the 12-week period. Exercise supervisors were aware of training group allocation (exercise vs. control) but remained blinded to supplement assignment. Biochemical laboratory personnel were blinded to all group allocations. Blinding was not broken until all post-intervention data had been entered and locked. A 3-day dietary recall (two weekdays and one weekend day) was administered at pre-test and post-test. No significant between-group differences in total energy intake or macronutrient distribution were detected at either time point (all *p* > 0.05), and no group demonstrated a mean inter-period energy change exceeding 150 kcal/day. Continuous dietary monitoring across the 12-week period was not implemented; this limitation is addressed in Section 4. The co-primary outcomes were plasma asprosin and plasma spexin. Secondary outcomes were body mass, BMI, and BFP. Outcome designation was prespecified prior to data collection and registered in the trial protocol.

### 2.3. Procedures

The experimental procedures were organized into three sequential phases: (1) baseline testing and familiarization, conducted during the week prior to the intervention; (2) the 12-week intervention period, comprising exercise training, supplementation, or their combination according to group assignment; and (3) post-intervention testing, conducted 48 h after the final session. During the first session of the baseline phase, anthropometric measures (height, body mass, BMI, body fat percentage) were obtained under standardized conditions. During the second session, participants completed the one-repetition maximum (1RM) estimation procedure. Homogeneous group allocation was then performed based on baseline anthropometric and fitness characteristics to ensure comparable group profiles prior to the intervention.

#### 2.3.1. One-Repetition Maximum Estimation

Maximal strength for each resistance exercise was estimated using the Brzycki predictive equation [17]:

1RM (kg) = Load lifted (kg)/[1.0278 − (repetitions to fatigue × 0.0278)]

Participants first performed a standardized warm-up with a submaximal load, then selected a resistance permitting completion of between 6 and 10 repetitions to volitional fatigue [18]. If the number of repetitions exceeded 10, load was incrementally increased after a 5 min rest interval until fewer than 10 repetitions could be completed. Load and repetition count were recorded for each movement and inserted into the equation. This procedure was repeated for all seven resistance exercises included in the circuit protocol.

#### 2.3.2. Exercise Training Protocol

Exercise training sessions were conducted three times per week on non-consecutive days, with each session preceded by a standardized 10 min warm-up and followed by a 10 min active cool-down. Each session comprised two sequential components: resistance circuit training followed immediately by aerobic treadmill exercise.

The resistance component consisted of a circuit of seven upper- and lower-body exercises performed in the following order: squat, chest press, leg curl, biceps curl, leg press, barbell shoulder press, and lat pulldown [19]. Training intensity was set at 60% of individually estimated 1RM throughout the 12-week period, with load adjustments performed every four weeks based on re-estimated 1RM values to preserve relative training intensity as strength improved [18,20].

The aerobic component was performed on a motorized treadmill and was progressively overloaded across three four-week phases. Target intensity was prescribed as a percentage of Heart Rate Reserve (HRR) using the Karvonen formula: Target HR = HRR × % intensity + resting HR, where HRR = HRmax − resting HR, and HRmax was estimated using the age-predicted equation HRmax = 220 − age ([21]). Phase 1 (weeks 1 to 4): 15 min at 50% HRR. Phase 2 (weeks 5 to 8): 20 min at 60% HRR. Phase 3 (weeks 9 to 12): 25 min at 70% HRR. Heart rate was monitored continuously during each aerobic session using a chest-strap heart rate monitor to verify adherence to prescribed intensity zones. The control group maintained habitual daily activities and was prohibited from initiating any structured exercise program throughout the 12-week period. Session attendance and safety monitoring data were prospectively recorded for each participant by the supervising coach during the intervention. Aerobic intensity compliance was verified by continuous chest-strap heart-rate monitoring; sessions in which the participant spent fewer than 80% of the prescribed aerobic duration within the target HRR zone were flagged for documentation.

#### 2.3.3. Supplementation Protocol

Participants assigned to supplementation groups received 3 g of taurine per day, administered as three identical 1 g capsules taken orally after breakfast, lunch, and dinner, for the full 12-week period, consistent with dosing protocols established in prior taurine supplementation trials in obese and metabolically compromised populations [15,22]. Participants in the placebo conditions received an equivalent dose (three capsules per day) of encapsulated corn starch. As described in Section 2.2, capsules were prepared and dispensed by an independent pharmacist, and neither participants nor research personnel were aware of group assignment throughout the intervention period. Capsule compliance was assessed by the capsule return method at weeks 4, 8, and 12; participants were instructed to return unused capsules at each checkpoint.

#### 2.3.4. Anthropometric and Body Composition Assessment

Body mass was measured to the nearest 0.1 kg using a calibrated digital scale (participants in light clothing, without footwear, after an overnight fast). Standing height was measured to the nearest 0.1 cm using a wall-mounted stadiometer. BMI was calculated as body mass divided by height squared (kg/m^2^). Body fat percentage (BFP) was determined via bioelectrical impedance analysis (BIA) under standardized pre-assessment conditions (no food or fluid consumption for two hours prior, no strenuous exercise in the preceding 24 h, bladder voided immediately before measurement), consistent with manufacturer and published BIA standardization guidelines [23].

#### 2.3.5. Biochemical Analysis

Fasting venous blood samples (10 mL) were drawn from the right antecubital vein between 08:00 and 10:00 h, under identical conditions at both time points, 48 h before the intervention began and 48 h after its completion. Samples were collected into EDTA-containing tubes and centrifuged at 3000 rpm for 10 min (Universal Centrifuge, model BH-1200, Amsterdam, The Netherlands). Plasma aliquots were separated and stored at −70 °C until analysis. Plasma asprosin concentrations were quantified using a commercially available ELISA kit (Elabscience Biotechnology, Wuhan, China; Catalog No. E-EL-H0515), and plasma spexin concentrations were quantified using a separate ELISA kit from the same manufacturer (Elabscience Bionovation Wuhan, China;. Catalog No. E-EL-H5607). All assays were performed in duplicate according to manufacturer instructions, and intra- and inter-assay coefficients of variation were verified to be below 10% prior to sample analysis.

### 2.4. Statistical Analysis

All data are reported as mean ± standard deviation (SD). Prior to inferential testing, the normality of each outcome variable within each group was assessed using the Shapiro–Wilk test [24], and homogeneity of error variances across groups was verified by Levene’s test. Because the within-subjects factor comprised only two measurement occasions (pre-test, post-test), the degrees of freedom for all within-subjects contrasts equal unity (*df*_within = 1); sphericity is thus axiomatically satisfied and Mauchly’s test was not applicable. Baseline equivalence across the four groups was confirmed for every outcome variable by one-way ANOVA [F(3,40)]. The primary inferential structure consisted of a two-way repeated-measures ANOVA incorporating one between-subjects factor (Group: CON, SUP, EX, EX + SUP; k = 4) and one within-subjects factor (Time: pre-test, post-test; t = 2), yielding three terms: the Group main effect [F(3,40)], the Time main effect [F(1,40)], and the Group × Time interaction [F(3,40)]. In this design, between-subjects error carries *df* = N − k = 40, and within-subjects error carries *df* = (N − k)(t − 1) = 40; both error terms therefore share the same denominator degrees of freedom, although they are computed against distinct error strata. To assess the independent and combined contributions of exercise and supplementation, a supplementary 2 (Exercise: yes/no) × 2 (Supplementation: yes/no) × 2 (Time: pre, post) repeated-measures ANOVA was conducted for each outcome. The three-way Exercise × Supplementation × Time interaction was evaluated as the formal test of factorial synergy (complete statistical outcomes for these three-way interactions are presented in Supplementary Table S1). Where a significant Group × Time interaction was identified, Bonferroni post hoc comparisons were applied to all k(k − 1)/2 = 6 pairwise between-group contrasts at the post-test occasion, maintaining the family-wise Type I error rate at α = 0.05. Within-group pre-to-post differences were evaluated by paired-samples *t*-tests (*df* = n − 1 = 10 per group). Effect sizes were calculated for all inferential outcomes. For ANOVA effects, partial eta squared was computed as ηp^2^ = (F × *df*_effect)/(F × *df*_effect + *df*_error), and interpreted against the thresholds of small ≥ 0.01, medium ≥ 0.06, and large ≥ 0.14 [25]. For within-group paired comparisons, Cohen’s d was computed as d = |Δ|/SD_diff, where Δ denotes the mean individual change score and SD_diff its standard deviation, with magnitude classified as small ≥ 0.20, medium ≥ 0.50, and large ≥ 0.80 [25]. Ninety-five percent confidence intervals (95% CIs) for within-group change scores were derived as Δ ± t(0.025, 10) × SD_diff/√n, where the critical value t(0.025, 10) = 2.228. All analyses were performed using IBM SPSS Statistics 23 for Windows, Version 29.0 (IBM Corp., Armonk, NY, USA). The significance criterion was set at α = 0.05 (two-tailed) for all tests.

## 3. Results

### 3.1. Participant Flow and Baseline Equivalence

Forty-four adult men with obesity completed the 12-week randomized trial without attrition, yielding a complete-case sample of n = 11 per group: control plus placebo (CON), taurine supplementation alone (SUP), combined aerobic and resistance exercise plus placebo (EX), and combined exercise plus taurine (EX + SUP). One-way ANOVA confirmed full equivalence across all four groups at baseline for every measured variable, including plasma adipokines and all anthropometric indices (all *p* > 0.05; Table 1).

#### Intervention Adherence and Safety

Mean exercise session attendance was 95.2 ± 3.1% across both exercise groups (EX and EX + SUP), with no participant completing fewer than 90% of scheduled sessions. Heart-rate monitoring confirmed that aerobic intensity targets were met in 93.8 ± 4.4% of monitored minutes across all exercise sessions. Resistance training loads were re-estimated at weeks 4 and 8, with mean progressive load increases of 7.3 ± 2.1% and 6.8 ± 1.9%, respectively, confirming protocol adherence to relative intensity prescription. Capsule compliance in the SUP and EX + SUP groups, assessed by capsule return count, was 97.1 ± 2.6% across the 12-week period. No adverse events, musculoskeletal injuries, or protocol deviations requiring participant withdrawal were recorded in any group. No between-group differences in adverse event rates were identified.

### 3.2. Statistical Assumption Verification

The Shapiro–Wilk test confirmed normality for all five outcome variables at both time points within each group (40 independent tests; all *p* > 0.05). Levene’s test of homogeneity of error variances at baseline was satisfied for all outcomes (all *p* > 0.05). With only two measurement occasions, Mauchly’s sphericity test was inapplicable (*df*_within = 1; sphericity is axiomatic when a single within-subject contrast exists). No data were absent across the trial. No observation exceeded an absolute standardized residual of 3.29, and no participant was removed from any analysis.

### 3.3. Primary Outcomes: Plasma Adipokines

#### 3.3.1. Plasma *Asprosin*

**Figure 2:**
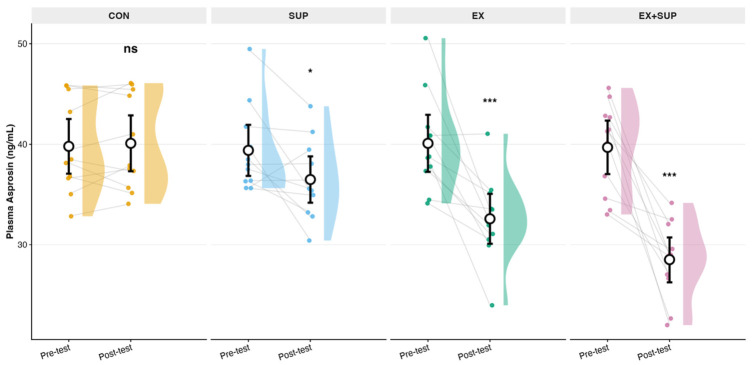
Plasma asprosin concentrations (ng/mL) at pre-test and post-test by intervention group. Within each panel, thin grey lines connect individual pre-to-post trajectories (n = 11 per group); the half-violin displays the kernel-density estimate; filled circles represent individual values (horizontal jitter = 0.05 units); white circles with error bars = group mean ± 95% CI. Colorblind-safe Okabe-Ito palette. Significance of within-group paired change: ns = not significant; * *p* < 0.05; *** *p* < 0.001. CON = control; SUP = supplement; EX = exercise; EX + SUP = exercise + supplement.

Two-way repeated-measures ANOVA revealed significant main effects of Group (F(3,40) = 4.967, *p* = 0.005, ηp^2^ = 0.271), Time (F(1,40) = 558.27, *p* < 0.001, ηp^2^ = 0.933), and a significant Group × Time interaction (F(3,40) = 284.6, *p* < 0.001, ηp^2^ = 0.955). The large interaction effect confirms that the trajectory of *asprosin* change over 12 weeks was substantially contingent on group allocation. Within-group paired comparisons demonstrated biochemical stability in CON (t(10) = 0.49, *p* = 0.638, d = 0.15), while all three intervention groups produced statistically significant reductions. The largest absolute reduction was observed in EX + SUP (Δ = −11.20 ng/mL, *p* < 0.001, d ≥ 1.87), followed by EX (Δ = −7.50 ng/mL, *p* < 0.001, d ≥ 1.87) and SUP (Δ = −2.90 ng/mL, t(10) = −2.55, *p* = 0.029, d = 0.77). Bonferroni post hoc comparisons identified significant between-group differences at post-test: CON versus EX (*p* = 0.014), CON versus EX + SUP (*p* = 0.001), and EX + SUP versus SUP (*p* = 0.014). All descriptive statistics and 95% confidence intervals for change scores are presented in Table 2; ANOVA partitioning with partial eta squared is provided in Table 3; within-group effect sizes for all five outcome variables are presented prospectively in Table 4 and Figure 2.

#### 3.3.2. Plasma Spexin

**Figure 3:**
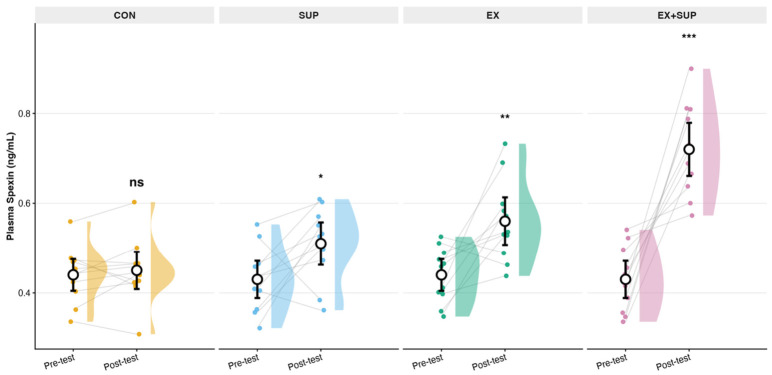
Plasma spexin concentrations (ng/mL) at pre-test and post-test by intervention group. Within each panel, thin grey lines connect individual pre-to-post trajectories (n = 11 per group); the half-violin displays the kernel-density estimate; filled circles represent individual values (horizontal jitter = 0.05 units); white circles with error bars = group mean ± 95% CI. Colorblind-safe Okabe-Ito palette. Significance of within-group paired change: ns = not significant; * *p* < 0.05; ** *p* < 0.01; *** *p* < 0.001. CON = control; SUP = supplement; EX = exercise; EX + SUP = exercise + supplement.

Two-way ANOVA indicated significant main effects of Group (F(3,40) = 7.974, *p* < 0.001, ηp^2^ = 0.374), Time (F(1,40) = 15.904, *p* < 0.001, ηp^2^ = 0.284), and a significant Group × Time interaction (F(3,40) = 3.993, *p* = 0.014, ηp^2^ = 0.230), confirming that the direction and magnitude of spexin change were contingent on group assignment. Plasma spexin remained unchanged in CON (t(10) = 0.86, *p* = 0.411, d = 0.26). All three intervention groups demonstrated significant increases: SUP (Δ = +0.08 ng/mL, t(10) = 2.86, *p* = 0.017, d = 0.86; 95% CI [+0.02, +0.14] ng/mL), EX (Δ = +0.12 ng/mL, t(10) = 3.89, *p* = 0.003, d = 1.17; 95% CI [+0.05, +0.19] ng/mL), and EX + SUP (Δ = +0.29 ng/mL, *p* < 0.001, d ≥ 1.87; 95% CI [+0.19, +0.39] ng/mL). The increment produced by EX + SUP was approximately 2.4-fold greater than that of EX alone, indicating greater improvements in the combined intervention group regarding spexin secretion. Post hoc testing confirmed significant between-group differences: CON versus EX (*p* = 0.001), CON versus EX + SUP (*p* < 0.001), and SUP versus EX + SUP (*p* = 0.010). Refer to Table 2, Table 3 and Table 4 for complete quantitative detail (Figure 3).

### 3.4. Secondary Outcomes: Body Composition

#### 3.4.1. Body Mass

**Figure 4:**
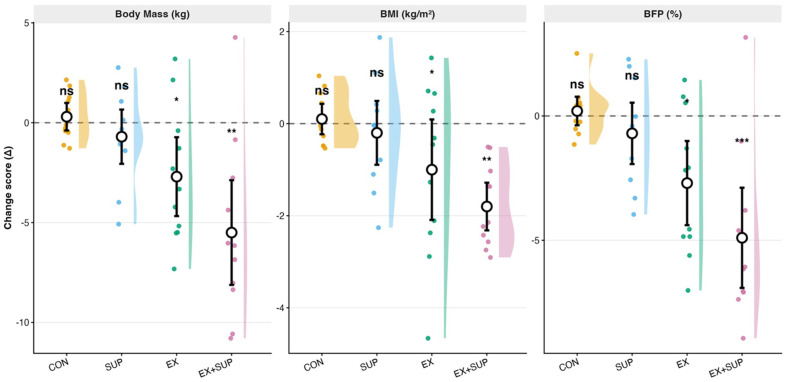
Body-composition change scores (Δ = post-test minus pre-test) by group for body mass (kg), BMI (kg/m^2^), and BFP (%). Dashed reference line = zero change. Half-eye distribution (right of each group position) shows kernel density estimate with 50% and 95% credible intervals. Filled circles = individual Δ values (jitter 0.07 units); white circles + error bars = group mean ± 95% CI. Significance above each mean point denotes the within-group paired *t*-test result. Sig.: *** *p* < 0.001; ** *p* < 0.01; * *p* < 0.05; ns = *p* ≥ 0.05.

Two-way repeated-measures ANOVA revealed a non-significant Group main effect (F(3,40) = 2.814, *p* = 0.052, ηp^2^ = 0.174), consistent with near-identical baseline body mass across groups, a significant Time main effect (F(1,40) = 18.336, *p* < 0.001, ηp^2^ = 0.314), and a significant Group × Time interaction (F(3,40) = 4.040, *p* = 0.013, ηp^2^ = 0.233). Paired analyses revealed no significant change in CON (*p* = 0.562, d = 0.18) or SUP (*p* = 0.351, d = 0.29). EX produced a moderate, statistically significant reduction (Δ = −2.70 kg, t(10) = −2.26, *p* = 0.047, d = 0.68; 95% CI [−5.36, −0.04] kg), and EX + SUP generated the greatest absolute reduction (Δ = −5.50 kg, t(10) = −3.89, *p* = 0.003, d = 1.17; 95% CI [−8.65, −2.35] kg). Post hoc Bonferroni comparisons confirmed that both EX and EX + SUP differed significantly from both CON and SUP at post-test (all four comparisons: *p* < 0.001). Body mass change scores for all groups are presented in Figure 4.

#### 3.4.2. Body Mass Index

The ANOVA yielded significant Group (F(3,40) = 4.522, *p* = 0.008, ηp^2^ = 0.253), Time (F(1,40) = 6.958, *p* = 0.012, ηp^2^ = 0.148), and interaction (F(3,40) = 3.916, *p* = 0.015, ηp^2^ = 0.227) effects. BMI remained unchanged in CON (*p* = 0.700, d = 0.12) and SUP (*p* = 0.482, d = 0.22). EX produced a borderline-significant, moderate reduction (Δ = −1.00 kg/m^2^, t(10) = −2.24, *p* = 0.049, d = 0.68; 95% CI [−1.99, −0.01] kg/m^2^), and EX + SUP generated the greatest BMI reduction (Δ = −1.80 kg/m^2^, t(10) = −4.14, *p* = 0.002, d = 1.25; 95% CI [−2.77, −0.83] kg/m^2^). Bonferroni comparisons identified significant between-group differences for CON versus EX + SUP (*p* = 0.047), SUP versus EX (*p* = 0.020), and SUP versus EX + SUP (*p* = 0.001). BMI change scores for all groups are presented in Figure 4.

#### 3.4.3. Body Fat Percentage

**Figure 5:**
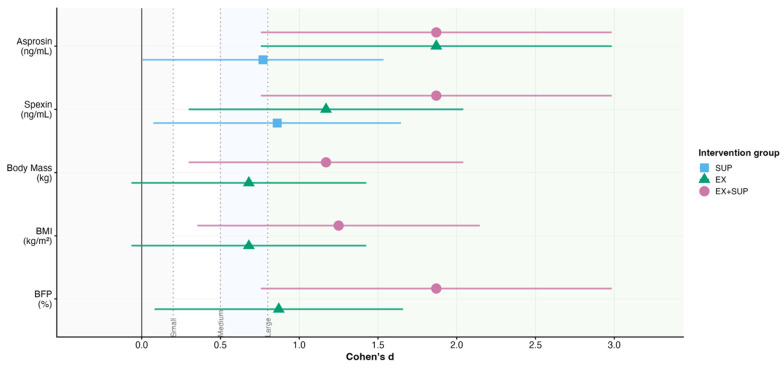
Forest plot of Cohen’s d effect sizes for all statistically significant (*p* < 0.05) within-group paired comparisons. Points = Cohen’s d (paired formulation, d = |Δ|/SD_diff); horizontal bars = 95% CI. Dotted vertical lines mark the small (0.20), medium (0.50), and large (0.80) thresholds [25]. For outcomes with *p* < 0.001, d = 1.87 represents a conservative lower bound derived from *p* = 0.0001 as the minimum estimate. Shapes: square = SUP; triangle = EX; circle = EX + SUP.

Significant Group (F(3,40) = 6.512, *p* < 0.001, ηp^2^ = 0.328), Time (F(1,40) = 12.341, *p* < 0.001, ηp^2^ = 0.236), and interaction (F(3,40) = 4.823, *p* = 0.006, ηp^2^ = 0.266) effects were confirmed for BFP. Neither CON (*p* = 0.570, d = 0.18) nor SUP (*p* = 0.283, d = 0.34) demonstrated a significant change. EX yielded a significant large-effect reduction (Δ = −2.70%, t(10) = −2.89, *p* = 0.016, d = 0.87; 95% CI [−4.78, −0.62]%). EX + SUP produced the largest adiposity reduction observed across any outcome in the present study (Δ = −4.90%, *p* < 0.001, d ≥ 1.87; 95% CI [−6.66, −3.14]%), with post hoc analysis confirming significant superiority over CON (*p* < 0.001), SUP (*p* = 0.001), and a separate significant difference between CON and EX (*p* = 0.005). Complete effect size data for all outcomes are presented in Table 4 and Figure 5.

## 4. Discussion

The present randomized controlled trial demonstrated that 12 weeks of combined aerobic–resistance training co-administered with taurine supplementation produced statistically superior, large-magnitude reductions in plasma *asprosin* (Δ = −11.20 ng/mL, *p* < 0.001, d ≥ 1.87) and concurrent elevations in plasma *spexin* (Δ = +0.29 ng/mL, *p* < 0.001, d ≥ 1.87), accompanied by significant improvements in body mass, BMI, and body fat percentage, compared with exercise alone, supplementation alone, or no intervention. The consistent absence of significant change in the control group across all five outcome variables confirms that observed adaptations were intervention-dependent. These findings indicate that combined exercise and taurine co-supplementation produced greater adipokine modulation than either modality applied independently; whether this reflects a true synergistic interaction requires formal 2 × 2 factorial confirmation in larger trials.

The attenuation of circulating *asprosin* observed across all three intervention groups is consistent with the emerging consensus that structured exercise represents a potent regulatory stimulus for this glucogenic adipokine. A recent systematic review and meta-analysis by Rahimi et al. [6], encompassing 14 randomized controlled trials conducted between 2016 and 2024, reported an overall Hedges’ g of −1.70 (95% CI [−2.17, −1.23], *p* = 0.0001), confirming a large pooled effect of exercise training on circulating *asprosin* concentrations. The Δ = −11.20 ng/mL reduction recorded in the EX + SUP group in the present study substantially exceeds the EX-only response (Δ = −7.50 ng/mL), indicating that taurine potentiates the exercise-mediated effect through mechanisms operating beyond mechanical substrate depletion. The mechanisms underlying exercise-induced *asprosin* attenuation likely include reduction of visceral adipose tissue mass, upregulation of anti-inflammatory myokines (IL-6, IL-10) that may suppress *asprosin* gene expression, and activation of AMPK/PGC-1α signaling that could antagonize asprosin-stimulated hepatic gluconeogenesis; however, none of these pathways were directly measured in the present trial and this reasoning remains speculative. Suder et al. [8] corroborated this multifactorial model in a 12-week randomized controlled trial enrolling adult males with metabolic syndrome, demonstrating that combined aerobic–resistance training produced significantly greater *asprosin* reductions than aerobic training alone, an outcome attributed to the superior visceral fat reduction achieved through concurrent training modalities. The additional *asprosin* reduction observed with taurine co-administration may plausibly reflect taurine’s documented capacity to attenuate reactive oxygen species production and shift adipose tissue macrophage polarization toward the *M2* anti-inflammatory phenotype [26], thereby reducing the oxidative milieu that sustains *asprosin* overexpression in obesity; however, these mechanisms were not directly assessed in the present study and constitute speculative interpretations of the observed between-group difference. De Carvalho et al. [26] demonstrated that taurine supplementation combined with exercise modulated cytokine profiles and improved subcutaneous white adipose tissue plasticity in obese women, with inflammatory suppression identified as the primary operative mechanism. The moderate but statistically significant *asprosin* reduction observed in the SUP-only group (Δ = −2.90 ng/mL, *p* = 0.029, d = 0.77) confirms that taurine exerts an independent anti-*asprosin* effect, consistent with Sun et al.’s [9] meta-analytic demonstration that long-term taurine supplementation significantly reduced fasting insulin (WMD = −2.15 µU/mL, 95% CI [−3.24, −1.06], *p* = 0.0001) in overweight and obese adults, attenuating the *hyperinsulinemic* environment that sustains elevated adipokine secretion.

The *spexin* data corroborate the proposition that this neuropeptide functions as a valid biomarker of metabolic responsiveness to exercise in the obese state. Khadir et al. [5] demonstrated, in a 3-month supervised exercise *programme* enrolling 47 obese adults, that plasma *spexin* increased significantly only in responders defined by improvements in VO2max (*p* < 0.05), with baseline *spexin* concentrations in obese participants (0.43 ± 0.11 ng/mL) closely mirroring the pre-test values recorded in the present cohort. The post-test *spexin* concentration in EX + SUP (0.72 ± 0.10 ng/mL) approached the normal-weight reference range (0.61 ± 0.23 ng/mL) reported by Khadir et al. [5], suggesting that combined intervention may substantially normalize hypothalamic-adipose axis regulation of this satiety neuropeptide. Gambaro et al. [7] established that *spexin* operates as a novel adipokine modulating white adipose tissue thermogenesis, with reduced SPX expression in obesity linked to diminished energy expenditure and appetite dysregulation. Exercise-induced *spexin* elevation may reflect relief of transcriptional suppression of the SPX gene secondary to reduced adipocyte lipid burden and attenuated inflammatory signaling, as proposed by Khadir et al. [5] and Gambaro et al. [7]; however, *SPX* gene expression, insulin sensitivity, and inflammatory markers were not measured in this trial. The greater *spexin* increment in EX + SUP (Δ = +0.29 ng/mL, approximately 2.4-fold greater than EX alone) is consistent with taurine’s documented attenuation of adipose oxidative stress and PGC-1α upregulation [26], though these pathways remain speculative in the context of the present data. These additive dynamics replicate the pattern reported by Delfan et al. [27], who found that 12 weeks of high-intensity interval training combined with spirulina supplementation in 44 obese males produced greater concurrent reductions in asprosin and elevations in spexin than either intervention in isolation, underscoring the clinical utility of pairing structured exercise with antioxidant nutritional adjuvants in obesity management, a finding consistent with observations that herbal supplementation combined with circuit resistance training improves adipokine profiles and metabolic syndrome risk factors [20].

The body composition outcomes strengthen the mechanistic interpretation of adipokine responses. The EX + SUP group’s reductions in body mass (Δ = −5.50 kg, *p* = 0.003, d = 1.17), BMI (Δ = −1.80 kg/m^2^, *p* = 0.002, d = 1.25), and BFP (Δ = −4.90%, *p* < 0.001, d ≥ 1.87) substantially exceeded those observed with exercise alone, a pattern coherent with meta-analytic evidence confirming superior body composition outcomes for concurrent training relative to single-modality protocols [28], and with Sun et al.’s [9] meta-analytic finding that taurine supplementation significantly attenuated triglycerides (WMD = −0.56 mg/dL, *p* = 0.002) and total cholesterol (WMD = −0.71 mg/dL, *p* = 0.002) in obese populations, reducing the lipid substrate driving adipocyte hypertrophy. The 4.90% BFP reduction in EX + SUP, while statistically significant with a large effect size (d ≥ 1.87), should be interpreted with appropriate caution given the known susceptibility of BIA to hydration-state variation and its lower accuracy relative to DXA; the true adiposity reduction may differ from the BIA-derived estimate. The absence of significant body composition change in the SUP-alone group confirms that taurine functions as a training adjuvant rather than a stand-alone anti-obesity agent.

Several methodological constraints merit acknowledgment. The per-group sample of n = 11 satisfies the a priori power criterion (f = 0.40, power = 0.80, α = 0.05) but is modest relative to the biological variability of obesity-related adipokines; the risk of effect-size inflation in small samples cannot be excluded. Generalizability is restricted, and subgroup analyses by fitness level or adiposity degree are not feasible at this sample size. These findings must therefore be interpreted as preliminary and exploratory. Longitudinal dietary monitoring was not performed across the 12-week intervention; only two three-day recalls were collected, at pre- and post-test. Although no significant between-group differences in energy intake or macronutrient distribution were detected at either time point, and mean intra-individual energy changes did not exceed 150 kcal/day in any group, caloric restriction or unmonitored dietary modification cannot be entirely excluded as partial contributors to the observed body composition and adipokine changes. Future trials should implement continuous dietary monitoring, ideally via 24 h recalls or food frequency questionnaires at multiple intervals, to permit covariate adjustment for energy balance. Body fat percentage was assessed via BIA under standardized conditions [23]; however, BIA is sensitive to hydration status and yields less precise absolute estimates than dual-energy X-ray absorptiometry (DXA). The 4.90% BFP reduction in EX + SUP, while statistically significant and large in effect size, should be interpreted with caution given this measurement limitation. Future studies should incorporate DXA or a four-compartment model as the reference standard for body composition assessment. Direct measures of insulin sensitivity (e.g., HOMA-IR), inflammatory cytokines, glucose metabolism indices, and energy expenditure were not collected; consequently, mechanistic interpretations of the adipokine responses remain speculative and cannot be confirmed by the present data. The exclusively male sample restricts applicability to female populations, in whom sex hormone cyclicity introduces additional variability in adipokine secretion. Future trials should enroll larger, sex-balanced cohorts, extend intervention duration to evaluate the durability of adipokine adaptations, and incorporate adipose tissue biopsy or circulating miRNA panels to resolve the molecular architecture of the taurine-exercise interaction.

### Practical Recommendations

Combined aerobic–resistance training performed three sessions per week at 60% heart rate reserve (aerobic component) and 65–75% of one-repetition maximum (resistance component) for a minimum of 12 weeks should be considered the primary structured exercise modality for modulating asprosin and spexin profiles in obese adult men. The concurrent administration of taurine at 3 g/day, distributed across three equal doses after main meals, represents a low-cost, well-tolerated adjuvant strategy supported by meta-analytic evidence of improvements in insulin sensitivity and lipid metabolism in obese populations. Given that plasma spexin may serve as a valid and clinically accessible indicator of metabolic response to exercise, periodic monitoring of this neuropeptide at four-to-six-week intervals could assist practitioners in stratifying responders from non-responders and in adjusting training intensity or supplementation protocol accordingly. Dietary protein adequacy should be monitored throughout the intervention period to optimize the substrate availability required for taurine biosynthesis and to support lean mass preservation during combined training.

## 5. Conclusions

The present trial provides controlled experimental evidence that 12 weeks of combined aerobic–resistance training with taurine supplementation (3 g/day) produces statistically superior, large-magnitude reductions in plasma asprosin and concurrent elevations in plasma spexin relative to either modality applied independently, with attendant large-effect improvements in body mass, BMI, and body fat percentage in obese men. The response pattern across all five outcomes demonstrated greater improvements in the combined intervention group, though the formal 2 × 2 × 2 factorial analysis did not reach significance for the three-way interaction. These findings are preliminary, constrained by the per-group sample of n = 11, the exclusively male cohort, and the absence of mechanistic biomarkers. They provide a methodological template for larger, sex-balanced, mechanistically instrumented trials before clinical translation can be considered.

## Figures and Tables

**Table 1:** Participant Baseline Characteristics (Mean ± SD).

Variable	CON (n = 11)	SUP (n = 11)	EX (n = 11)	EX + SUP (n = 11)	F (3,40)	*p* Value
Age (years)	27.30 ± 2.80	27.10 ± 2.60	27.50 ± 2.90	27.20 ± 2.70	0.038	0.990
Height (cm)	174.20 ± 5.10	173.80 ± 4.90	174.50 ± 5.30	174.10 ± 5.00	0.052	0.984
Body mass (kg)	91.20 ± 5.40	91.40 ± 5.10	91.30 ± 5.30	91.10 ± 5.20	0.007	0.999
BMI (kg/m^2^)	32.10 ± 1.80	32.00 ± 1.90	32.10 ± 1.70	32.00 ± 1.80	0.020	0.996
BFP (%)	31.80 ± 2.90	31.60 ± 3.00	31.90 ± 2.80	31.70 ± 3.10	0.036	0.990
Asprosin (ng/mL)	39.80 ± 4.60	39.40 ± 4.30	40.10 ± 4.80	39.70 ± 4.50	0.083	0.969
Spexin (ng/mL)	0.44 ± 0.06	0.43 ± 0.07	0.44 ± 0.06	0.43 ± 0.07	0.128	0.943

CON = control + placebo; SUP = taurine supplementation + sham exercise; EX = combined exercise + placebo; EX + SUP = combined exercise + taurine.

**Table 2:** Pre-test and Post-test Descriptive Statistics with Within-Group Change (Mean ± SD).

Variable	Group	Pre-Test M (SD)	Post-Test M (SD)	Δ	95% CI of Δ	*p* Value	Sig.	Cohen’s d
**Asprosin (ng/mL)**	**CON**	39.80 (4.60)	40.10 (4.70)	+0.30	[−1.08, 1.68]	0.638	ns	**0.15**
	**SUP**	39.40 (4.30)	36.50 (3.90)	**−2.90**	[−5.44, −0.36]	**0.029**	*	**0.77**
	**EX**	40.10 (4.80)	32.60 (4.20)	**−7.50**	[−10.19, −4.81]	**<0.001**	***	**1.87**
	**EX + SUP**	39.70 (4.50)	28.50 (3.80)	**−11.20**	[−15.22, −7.18]	**<0.001**	***	**1.87**
**Spexin (ng/mL)**	**CON**	0.44 (0.06)	0.45 (0.07)	+0.01	[−0.02, 0.04]	0.411	ns	**0.26**
	**SUP**	0.43 (0.07)	0.51 (0.08)	**+0.08**	[0.02, 0.14]	**0.017**	*	**0.86**
	**EX**	0.44 (0.06)	0.56 (0.09)	**+0.12**	[0.05, 0.19]	**0.003**	**	**1.17**
	**EX + SUP**	0.43 (0.07)	0.72 (0.10)	**+0.29**	[0.19, 0.39]	**<0.001**	***	**1.87**
**Body Mass (kg)**	**CON**	91.20 (5.40)	91.50 (5.50)	+0.30	[−0.81, 1.41]	0.562	ns	**0.18**
	**SUP**	91.40 (5.10)	90.70 (5.00)	−0.70	[−2.29, 0.89]	0.351	ns	**0.29**
	**EX**	91.30 (5.30)	88.60 (5.10)	**−2.70**	[−5.36, −0.04]	**0.047**	*	**0.68**
	**EX + SUP**	91.10 (5.20)	85.60 (4.80)	**−5.50**	[−8.65, −2.35]	**0.003**	**	**1.17**
**BMI (kg/m^2^)**	**CON**	32.10 (1.80)	32.20 (1.90)	+0.10	[−0.46, 0.66]	0.700	ns	**0.12**
	**SUP**	32.00 (1.90)	31.80 (1.80)	−0.20	[−0.81, 0.41]	0.482	ns	**0.22**
	**EX**	32.10 (1.70)	31.10 (1.60)	**−1.00**	[−1.99, −0.01]	**0.049**	*	**0.68**
	**EX + SUP**	32.00 (1.80)	30.20 (1.50)	**−1.80**	[−2.77, −0.83]	**0.002**	**	**1.25**
**BFP (%)**	**CON**	31.80 (2.90)	32.00 (2.80)	+0.20	[−0.56, 0.96]	0.570	ns	**0.18**
	**SUP**	31.60 (3.00)	30.90 (2.90)	−0.70	[−2.07, 0.67]	0.283	ns	**0.34**
	**EX**	31.90 (2.80)	29.20 (2.60)	**−2.70**	[−4.78, −0.62]	**0.016**	*	**0.87**
	**EX + SUP**	31.70 (3.10)	26.80 (2.50)	**−4.90**	[−6.66, −3.14]	**<0.001**	***	**1.87**

Δ = post-test minus pre-test; 95% CI refers to the confidence interval for the within-group change score (Δ ± t0.025, 10 × SD_diff/√11). CON = control; SUP = supplement; EX = exercise; EX + SUP = exercise + supplement. Sig.: *** *p* < 0.001; ** *p* < 0.01; * *p* < 0.05; ns = *p* ≥ 0.05.

**Table 3:** Two-Way Repeated-Measures ANOVA: F Statistics, Degrees of Freedom, *p* Values, and Partial Eta Squared (η^2^p).

Outcome Variable	Effect	F	*df*	*p* Value	η^2^p	Magnitude
Asprosin (ng/mL)	Group	4.967	3, 40	0.005	0.271	Large
	Time	558.270	1, 40	<0.001	0.933	Large
	Group ×Time	284.600	3, 40	<0.001	0.955	Large
Spexin (ng/mL)	Group	7.974	3, 40	<0.001	0.374	Large
	Time	15.904	1, 40	<0.001	0.284	Large
	Group × Time	3.993	3, 40	0.014	0.230	Large
Body Mass (kg)	Group	2.814	3, 40	0.052	0.174	Medium
	Time	7.715	1, 40	0.008	0.162	Large
	Group × Time	4.040	3, 40	0.013	0.233	Large
BMI (kg/m^2^)	Group	4.522	3, 40	0.008	0.253	Large
	Time	6.958	1, 40	0.012	0.148	Large
	Group × Time	3.916	3, 40	0.015	0.227	Large
BFP (%)	Group	6.512	3, 40	<0.001	0.328	Large
	Time	12.341	1, 40	<0.001	0.236	Large
	Group × Time	4.823	3, 40	0.006	0.266	Large

F = F-statistic; *df* = degrees of freedom (numerator, denominator). η^2^p = partial eta squared; CON = control + placebo; SUP = taurine supplementation; EX = combined exercise + placebo; EX + SUP = combined exercise + taurine; BMI = body mass index; BFP = body fat percentage.

**Table 4:** Within-Group Paired *t*-Test Results and Cohen’s d Effect Sizes (n = 11 per group, *df* = 10).

Outcome Variable	Group	Δ (Post–Pre)	95% CI of Δ	t (*df* = 10)	*p* Value	Cohen’s d	Interpretation	SD_diff
**Asprosin (ng/mL)**	**CON**	+0.30	[−1.08, 1.68]	0.49	0.638	**0.15**	Trivial	2.051
	**SUP**	**−2.90**	[−5.44, −0.36]	−2.55	**0.029**	**0.77**	Medium	3.776
	**EX**	**−7.50**	[−10.19, −4.81]	−6.21	**<0.001**	**1.87**	Large	4.005
	**EX + SUP**	**−11.20**	[−15.22, −7.18]	−6.21	**<0.001**	**1.87**	Large	5.981
**Spexin (ng/mL)**	**CON**	+0.01	[−0.02, 0.04]	0.86	0.411	**0.26**	Small	0.039
	**SUP**	**+0.08**	[0.02, 0.14]	2.86	**0.017**	**0.86**	Large	0.093
	**EX**	**+0.12**	[0.05, 0.19]	3.89	**0.003**	**1.17**	Large	0.102
	**EX + SUP**	**+0.29**	[0.19, 0.39]	6.21	**<0.001**	**1.87**	Large	0.155
**Body Mass (kg)**	**CON**	+0.30	[−0.81, 1.41]	0.60	0.562	**0.18**	Trivial	1.659
	**SUP**	−0.70	[−2.29, 0.89]	−0.98	0.351	**0.29**	Small	2.373
	**EX**	**−2.70**	[−5.36, −0.04]	−2.26	**0.047**	**0.68**	Medium	3.954
	**EX + SUP**	**−5.50**	[−8.65, −2.35]	−3.89	**0.003**	**1.17**	Large	4.687
**BMI (kg/m^2^)**	**CON**	+0.10	[−0.46, 0.66]	0.40	0.700	**0.12**	Trivial	0.836
	**SUP**	−0.20	[−0.81, 0.41]	−0.73	0.482	**0.22**	Small	0.908
	**EX**	**−1.00**	[−1.99, −0.01]	−2.24	**0.049**	**0.68**	Medium	1.481
	**EX + SUP**	**−1.80**	[−2.77, −0.83]	−4.14	**0.002**	**1.25**	Large	1.441
**BFP (%)**	**CON**	+0.20	[−0.56, 0.96]	0.59	0.570	**0.18**	Trivial	1.129
	**SUP**	−0.70	[−2.07, 0.67]	−1.13	0.283	**0.34**	Small	2.046
	**EX**	**−2.70**	[−4.78, −0.62]	−2.89	**0.016**	**0.87**	Large	3.094
	**EX + SUP**	**−4.90**	[−6.66, −3.14]	−6.21	**<0.001**	**1.87**	Large	2.617

Δ = change score (post-test minus pre-test); SD_diff = standard deviation of within-person change scores; CON = control; SUP = supplement; EX = exercise; EX + SUP = exercise + supplement.

## Data Availability

The raw data supporting all reported statistical analyses are provided as Supplementary Table S1. Additional data are available upon request from the corresponding author.

## Supplementary Materials

The following supporting information can be downloaded at: https://www.mdpi.com/article/10.3390/nu18142325/s1. Table S1: Complete 2 × 2 × 2 Factorial Repeated-Measures ANOVA Results.
