# Supplementary material for: Combined Aerobic–Resistance Training and Taurine Supplementation Reduce Asprosin and Elevate Spexin in Men with Obesity: A 12-Week Supplement-Blinded, Randomized Controlled Trial
Source: Nutrients. 2026 Jul 16;18(14):2325. doi: 10.3390/nu18142325 (PMC13415041; doi:10.3390/nu18142325)
Supplement: Supplementary file 1 [file nutrients-18-02325-s001.zip › nutrients-4381929-supplementary.pdf]

Supplementary Table S1. Complete 2 × 2 × 2 Factorial Repeated-Measures ANOVA Results.

Outcome Variable	Effect	F (1, 40)	*p*-Value	Partial Eta Squared (η_p^2)
Asprosin (ng/mL)	Time × Exercise × Supplement	1.333	0.255	0.032
Spexin (ng/mL)	Time × Exercise × Supplement	2.136	0.152	0.051
Body Mass (kg)	Time × Exercise × Supplement	0.951	0.335	0.023
BMI (kg/m^2)	Time × Exercise × Supplement	0.469	0.498	0.012
Body Fat Percentage (%)	Time × Exercise × Supplement	0.740	0.395	0.018

Note: The absence of statistical significance ($p \geq 0.05$) across all variables for the three-way interaction confirms that while the combined intervention (EX+SUP) produced the greatest absolute improvements, the interaction between exercise and taurine does not meet the formal threshold for an additive or synergistic effect.
